# A capacity strengthening model toward self-reliant and sustainable one-health workforce in six East African Community Partner States

**DOI:** 10.3389/fpubh.2025.1636817

**Published:** 2025-07-24

**Authors:** Julien A. Nguinkal, Florian Gehre, Hakim I. Lagu, Emmanuel Achol, Eric Nzeyimana, John N. Kiiru, Joseph Nyandwi, Gregory W. Dumo, Nyambura Moremi, Susan N. Nabadda, Isabelle Mukagatare, Andrea Molina, Jürgen May, Muna Affara

**Affiliations:** ^1^Department of Infectious Disease Epidemiology, Bernhard-Nocht Institute for Tropical Medicine, Hamburg, Germany; ^2^Health Department, East African Community (EAC), Arusha, Tanzania; ^3^Department of Disease Surveillance and Epidemic Response, Ministry of Health, Nairobi, Kenya; ^4^National Reference Laboratory, National Institute of Public Health, Bujumbura, Burundi; ^5^National Public Health Laboratory, Ministry of Health, Juba, South Sudan; ^6^National Public Health Laboratory, Ministry of Health, Dar es Salaam, Tanzania; ^7^Central Public Health Laboratories, National Health Laboratories, Ministry of Health, Kampala, Uganda; ^8^Biomedical Services Department, Rwanda Biomedical Centre, Kigali, Rwanda; ^9^German Center for Infection Research (DZIF), Hamburg-Borstel-Lübeck-Riems, Hamburg, Germany; ^10^Tropical Medicine II, University Medical Center Hamburg-Eppendorf (UKE), Hamburg, Germany

**Keywords:** genomic surveillance, bioinformatics capacity building, Training-of-Trainers (ToT), East African Community (EAC), National Public Health Laboratories (NPHLs), antimicrobial resistance (AMR), One Health

## Abstract

The burden of infectious diseases and antimicrobial resistance (AMR) in Africa highlights the critical need for strengthened genomic surveillance capacities that are embedded within the national public health framework. In the East African Community (EAC), this challenge is compounded by limited infrastructure and insufficient workforce capacity in bioinformatics and genomics, particularly within National Public Health Laboratories (NPHLs). This paper describes the implementation of a regional capacity-building initiative based on a multi-phase Training-of-Trainers (ToT) model across six EAC Partner States. Anchored in a One Health framework, the initiative focused on equipping public health professionals within NPHLs with practical skills in pathogen genomics, AMR analysis, and bioinformatics workflows, while also supporting the institutionalization of standardized procedures and tools. Through modular training, in-country cascade sessions, and structured mentorship, the program enabled integration of genomic approaches into public health surveillance activities. Despite infrastructural and operational constraints, the initiative supported measurable gains in applied proficiency, routine use of genomics tools in surveillance tasks, and regional coordination on pathogen data analysis. This case study outlines the program's design, implementation, and observed outcomes, and offers a transferable framework for workforce and systems development in low-resource settings. This experience contributes to ongoing global discussions on equitable genomic surveillance and preparedness by demonstrating how structured, context-specific training can support sustainable adoption of genomics within national public health institutions.

## Introduction

The East African Community (EAC), like other African regions, is disproportionately burdened by infectious diseases, such as endemic malaria, tuberculosis, and HIV/AIDS, as well as emerging threats like viral hemorrhagic fevers, arboviruses, and antimicrobial resistance (AMR) (1, 2). Over the past two decades, the EAC region has experienced the highest frequency of disease outbreaks in Africa, averaging 15 reported outbreak events per year (3, 4). These persistent public health challenges underscore the pressing need for robust and context-specific genomic tools and capacities that enable timely disease detection and containment at the source. In its global surveillance strategy, the World Health Organization (WHO) emphasizes the importance of effective national genomic surveillance capacities as a cornerstone of a robust global health architecture, encompassing preparedness, response, and resilience to global health threats (5, 6). Pathogen genomics and bioinformatics have emerged as powerful tools in this endeavor, allowing rapid pathogen identification, outbreak tracking, and the development of effective interventions (7, 8). When integrated with epidemiological information and clinical metadata, next-generation sequencing (NGS) of pathogens provides invaluable insights for public health research, guiding evidence-based interventions, treatment strategies, and outbreak management (9, 10).

Despite these potential benefits, the widespread adoption and implementation of advanced genomics tools in low-resource settings still face significant roadblocks. The high costs associated with establishing genomic infrastructure, coupled with the need for specialized bioinformatics skills, often render these technologies inaccessible to medical and public health laboratories in high-burden regions like the EAC. For instance, ~97% of AMR-priority pathogens isolated in the EAC over the past decade have been sequenced and analyzed outside Africa, primarily in Europe and North America (11). While there has been increasing investment in establishing local sequencing capacity through infrastructure development, the commitment and resources for establishing bioinformatics capacity have often lagged behind. As a result, even when samples are sequenced locally, countries still rely on external support for analysis. This reliance on external entities for genomic analysis is largely due to limited digital infrastructure, technical constraints, scarce financial resources, and a shortage of trained public health personnel in pathogen genomics and bioinformatics (11, 12). This imbalance significantly undermines the ability of local public health agencies to formulate timely and effective outbreak mitigation strategies, and weakens global health preparedness efforts against future pandemics (13, 14).

In response, the EAC Secretariat, in partnership with the Bernhard–Nocht Institute of Tropical Medicine (BNITM), has developed and implemented a regional capacity-building initiative to enhance genomic surveillance capabilities within the EAC. This initiative features a multi-phased “Training of Trainers” (ToT) concept focusing on NGS-based AMR surveillance, viral bioinformatics, genomic epidemiology, and high-performance computing for large-scale pathogen data analysis. The collaborative effort aims to empower National Public Health Laboratories (NPHLs) within the Ministries of Health by providing the tools and expertise necessary for self-reliant pathogen and disease surveillance. This approach aims to strengthen the region's capacity to detect and respond to emerging public health threats and aligns with the WHO's 10-year global genomic surveillance strategy for pathogens with pandemic and epidemic potential (6).

Here, we report in this case-study on the achievements, challenges, lessons learned, and future directions of the ToT program implemented in the EAC region. We detail the program's design, implementation, and outcomes, emphasizing the importance of building local-to-regional expertise for a sustainable pathogen genomics landscape. Furthermore, we share insights that can inform similar efforts across Africa, contributing to the global discourse on integrating genomic tools into clinical and public health practices at both national and regional levels. Such efforts are crucial for developing equitable and context-specific pathogen genomics capacities, ultimately contributing to the global health surveillance and preparedness framework (15, 16).

## Regional context

The EAC is a regional intergovernmental organization that, in 2022, expanded to include eight Partner States including Burundi, DR Congo, Kenya, Rwanda, Somalia, South Sudan, Uganda, and Tanzania, with its headquarters in Arusha, Tanzania. The EAC is home to an estimated 331 million citizens, of which over 30% is urban population. These countries share socio-ecological dynamics and similar disease ecologies, making regional collaboration essential for outbreak control. The EAC Secretariat actively supports public health integration among Partner States, including a rapid response mobile laboratory network and several initiatives to strengthen NPHLs through equipment upgrades and training programs. Despite these efforts, substantial disparities in genomic capabilities persist across the EAC's Partner States (12). For example, only a few countries (e.g., Kenya, Tanzania, Uganda) in the region have well-established in-country sequencing and bioinformatics facilities; most other Partner States still rely on partnerships with external institutions for bioinformatics analysis (17, 18). A regional assessment highlighted the need to build local capacity in microbial pathogen sequencing and data analysis within NPHLs, enabling them to conduct these activities independently and enhance the timeliness and sustainability of surveillance and outbreak response across the EAC (12).

In this context, the ToT program was co-led by the EAC Secretariat and BNITM (Hamburg, Germany), with support from national health ministries and public health laboratories of the Partner States (19). Six Partner States – Burundi, Rwanda, Kenya, Tanzania, South Sudan, and Uganda—participated in the first implementation (Box 1). Each country nominated three candidates (laboratory scientists or epidemiologists working on public health surveillance), from which two were selected based on basic genomics experience and potential to serve as trainers. Selecting candidates embedded in the health system ensured direct pathways for knowledge transfer and alignment with national public health priorities.

Box 1 **Objectives of the EAC's Bioinformatics ToT concept.**

**Overall aim**

The overarching objective of the Bioinformatics ToT concept is to build equitable and sustainable capacities for genomic surveillance and bioinformatics within the region.

**Specific objectives**

a. Strengthen local and regional capacity: Equip national public health institutions with the necessary skills, infrastructure and resources for pathogen sequencing and data analysis.
b. Promote integration of NGS: Foster the integration of NGS into broader public health laboratory networks, including the established RRMLs network.
c. Foster regional collaboration: Enhance regional collaboration among public health laboratories to address cross-border health threats through data, experience and knowledge sharing.
d. Stimulate a multiplier effect: Build a cadre of professionals who can train others within their respective institutions, establishing a country-led and sustained dissemination of skills and expertise.

**Strategic relevance**

Collectively, this ToT concept is particularly relevant in the resource-limited context of the EAC in different aspects. First, by training professionals already embedded within local health systems, the program ensures that expertise remains within the region and can be directly applied to the specific challenges faced by each country. Second, the ToT model also creates a multiplier effect that fosters a self-reliant and sustained public health workforce. Additionally, it supports the overarching goal of regional harmonization of genomic surveillance efforts through a network of trained professionals who can collaborate and share best practices.

## Programmatic elements

**Figure 1:**
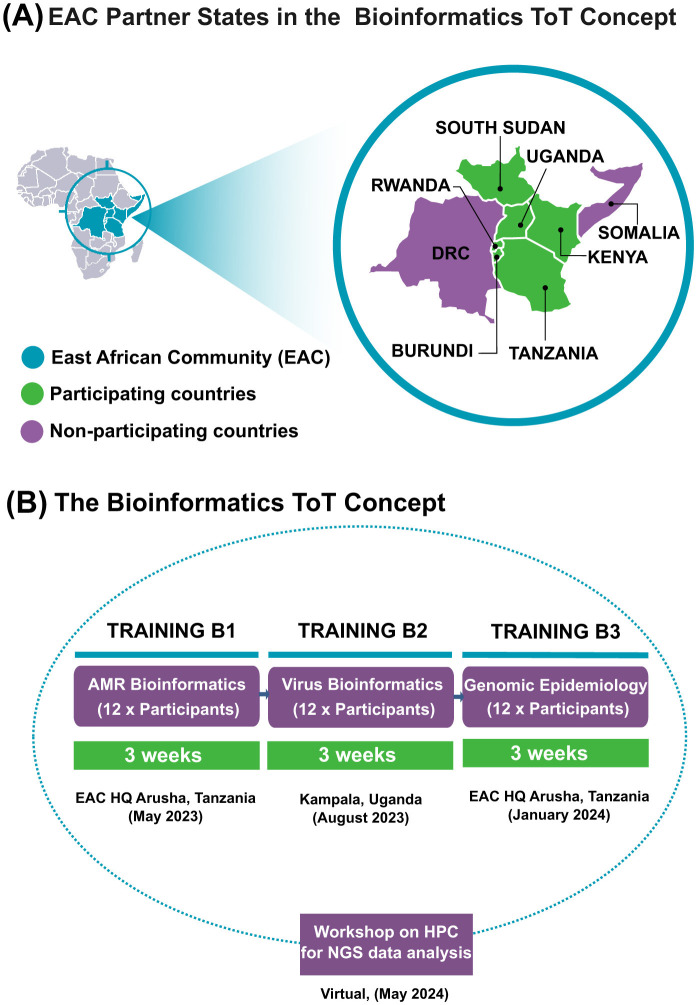
Overview of the Bioinformatics Training ofs Trainers (ToT) program in the East African Community (EAC). **(A)** EAC Partner States participating in the ToT program. **(B)** Structure and timeline of the three training phases (AMR bioinformatics, viral bioinformatics, and genomic epidemiology) and the workshop on high-performance computing (HPC) for NGS data analysis.

The capacity-strengthening initiative was built around three strategic pillars: (1) upgrading infrastructure (sequencing and computing resources, data pipelines, and connectivity), (2) developing the workforce via practical training, and (3) fostering institutional support (through stakeholder engagement and networks). We aligned the curriculum with EAC public health priorities: antimicrobial resistance (AMR) surveillance, viral haemorrhagic fever diagnostics, genomic-driven outbreak response, and genomic epidemiology (Figure 1). Training sessions were delivered through a Training-of-Trainers (ToT) model, empowering selected professionals to cascade skills to their colleagues, ensuring local ownership and sustainability.

**Figure 2:**
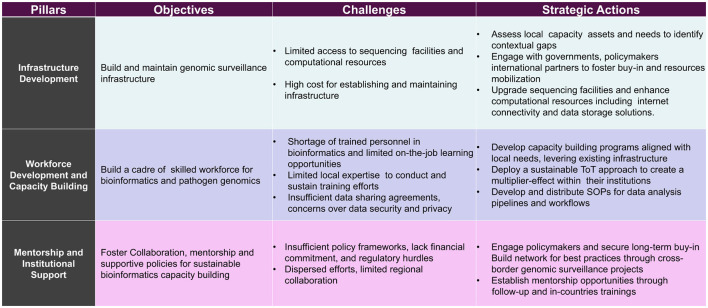
Proposed Bioinformatics capacity building framework. The figure presents a high-level framework for establishing sustainable bioinformatics capacity, particularly in resource-constrained environments. The framework builds on three pillars: Infrastructure Development (1), Workforce Development and Capacity Building (2), and Mentorship and Institutional Support (3). Each pillar outlines key objectives, challenges, and strategic actions to address those challenges. The framework emphasizes a holistic approach that combines technical infrastructure, skilled personnel, and supportive policies to enable sustainable and resilient pathogen genomics and bioinformatics capabilities.

- Targeted curriculum aligned to regional priorities. Modules focused on regional public health needs: Phase 1 covered *AMR bioinformatics* (NGS methods, genome assembly, and resistance gene identification); Phase 2 covered *viral genomics* (virus sequencing data analysis, mutation tracking, and outbreak phylogenetics); Phase 3 integrated *genomic epidemiology* (merging sequence data with outbreak metadata, phylogeography, and transmission analysis). A 1-day workshop on high-performance computing (HPC) introduced analysis of large datasets (Figure 2).
- Small cohort and hands-on approach. Only 12–14 participants were trained together (two per country). This limited size allowed personalized instruction and interactive learning, as opposed to mass workshops. Trainers could adapt pacing to participants' backgrounds. Standard Operating Procedures (SOPs) and reproducible workflows were developed to facilitate hands-on practice and ensure consistency in procedures.
- Training-of-Trainers and sustainability. After the core modules, each trainee (now ToT) was expected to lead *in-country* workshops and refresher sessions for colleagues at their home institutions. This cascading model was built into the program from the start, extending the reach of training beyond the initial group and mitigating knowledge loss from staff turnover. Local mentorship and continued support (e.g., regular virtual check-ins) were implemented to reinforce skills application.
- Infrastructure support. Recognizing limited technical capacity in some labs, trainers provided offline solutions: large reference databases and analysis pipelines were preloaded onto external drives. Each participating NPHL received baseline hardware (e.g., laptops and benchtop sequencers) to enable sequencing runs and bioinformatics processing.

To ensure sustainability, in-country training was embedded as a core part of the program. After centralized training, each participant was mandated to lead local sessions within their public health institutions. This cascade training model helps retain capacity despite staff turnover by institutionalizing SOPs and workflows. Refresher trainings are underway in Burundi, Tanzania and Uganda, and a regional monitoring system tracks adoption and integration across countries. However, sustainability depends not only on trained individuals but on embedding capacity within national systems. Aligning the program with health strategies, leadership engagement, and ongoing funding is essential to maintain the gains of this effort beyond the initial program implementation.

## Methodology and implementation scope

The program was implemented in three core phases, each lasting 3 weeks and focusing on a distinct area of expertise (Figure 2). The full roll-out spanned ~20 months, from May 2023 to January 2025. Phase 1 (AMR bioinformatics) was delivered in May 2023, Phase 2 (viral bioinformatics) in August 2023, and Phase 3 (genomic epidemiology and high-performance computing) across two sessions in January and May 2024. Follow-up in-country cascade trainings—led by ToT participants—were conducted in Burundi (October 2024), Tanzania (March 2025), Rwanda (April 2025), and South Sudan (May 2025). Refresher trainings also commenced in Burundi and Uganda in early 2025 to reinforce practical application and sustainability.

Phase 1: AMR bioinformatics: the first phase covered AMR bioinformatics, encompassing NGS technologies, sequencing techniques, and data analysis. Emphasis was placed on quality control, genome assembly, identification of resistance genes, and antimicrobial resistance (AMR) profiling.

Phase 2: Viral bioinformatics: the second phase focused on viral bioinformatics, equipping participants with the skills to analyze viral sequencing data, identify mutations associated with drug resistance or virulence, and track viral evolution. Participants learned to leverage these tools for effective viral surveillance and outbreak management.

Phase 3: Genomic and Epidemiological Integration: the third phase focused on integrating genomic and epidemiological data to investigate outbreaks, track transmission patterns, and inform public health interventions. This phase also included the deployment of public health bioinformatics workflows, enabling participants to apply their knowledge in practical genomic surveillance scenarios. Complementing these three phases was a 1-day workshop designed to demonstrate how to leverage HPC resources for large-scale pathogen NGS data analysis. This workshop equipped participants with the necessary skills to handle complex datasets and perform comprehensive analyses using advanced computational tools.

Refresher and in-country training for sustainability: to ensure the sustainability of these capacity-building efforts, subsequent refresher and in-country training sessions are expected to be conducted in each participating EAC's Partner State. In these sessions, the ToTs will impart their knowledge and skills to other staff members within their respective institutions. This approach not only extends the reach of the initial training sessions but also creates a resilient framework for skills retention within the institutions, even in the event of personnel turnover.

The training methodology combined theoretical lectures, hands-on exercises, and case studies using real-world datasets. Participants could apply the methods and skills they learned in practical scenarios, enhancing their understanding and proficiency. Particular emphasis was placed on guiding the participants through the entire bioinformatics workflow, from developing analysis scripts to data visualization and interpretation. This end-to-end exposure enabled them to gain experience in addressing potential errors or issues that may arise when working with real-world pathogen datasets, thereby refining their problem-solving skills, which are crucial in bioinformatics. At the end of each phase, we assessed skills through pre- and post-training quizzes and surveys.

## Measurable outcomes

We evaluated outcomes through pre- / post-assessments, usage metrics, and participant surveys. Key impacts included:

- Rapid skill gains. Participants' proficiency assessment showed marked improvement. On average, confidence scores rose by 26% after Phase 1 and by 16% after Phase 2. The steeper gains in the first phase likely reflect building foundational skills; Phase 3 showed consolidation of advanced skills. This indicates effective learning across modules (Supplementary Figure S2).
- Increased adoption of bioinformatics tools. Before training, only 20% of participants had been using bioinformatics tools (e.g., command-line pipelines) on a weekly basis. After training, 40% reported using the tools weekly, and 80% used them monthly. Trainees began incorporating analysis pipelines into routine surveillance tasks. For instance, several countries established monthly sequencing runs for priority pathogens. This uptake suggests the training translated into practice, not just knowledge.
- Applied research and surveillance projects. Trainees reported applying their new skills in ongoing projects. Fifty per cent said they used genomics tools in 1–2 active projects and 33% in 3–4 projects. These projects ranged from characterizing outbreak strains (e.g., cholera in Tanzania, Burundi) to AMR surveillance. The increasing integration of NGS data into these activities demonstrates enhanced research capacity in NPHLs. In-country implementation following the ToT program has already yielded concrete outcomes. In Burundi, trained staff led sequencing and genomic analysis for outbreak response efforts (19). Similarly, in Tanzania, ToTs sequenced and analyzed Marburg virus strains during a national response. In-country wet lab and bioinformatics trainings have also been successfully conducted by ToTs in Uganda, Tanzania, Rwanda, and Burundi, expanding institutional capacity beyond the initial trainees and reinforcing routine integration of genomics into national surveillance activities.
- Standardized workflows and SOPs. A lasting outcome was the development of shared Standard Operating Procedures (SOPs) for sequencing and analysis. All trainees received documented pipelines for AMR gene analysis, viral genome assembly, and phylogenetic reconstruction. Having common protocols improved result consistency across labs and eased troubleshooting. These resources also serve as teaching materials for future trainings, facilitating scale-up.
- Strengthened regional network. The program fostered the development of a regional genomic surveillance network by training representatives from six EAC countries together. This approach strengthened both personal and institutional linkages, facilitating cross-border collaboration in sequencing, data sharing, and outbreak investigations. Trainees established communication channels to exchange technical support, troubleshoot challenges, and coordinate regional projects. This network promotes an integrated response to infectious disease outbreaks, enabling countries to jointly tackle shared public health threats. The ToT model has thus laid the foundation for a sustainable regional workforce capable of supporting national and cross-border genomic surveillance activities. This aligns with global health security priorities, particularly WHO's objectives for strengthening regional genomic capabilities, and enhances the EAC's preparedness to detect and respond to public health emergencies (6).

In response to the need for broader access, selected SOPs and training materials have already been made publicly available through a dedicated GitHub repository (link provided in the **Data Availability** section). These include the full training schedule, module outlines, hands-on exercises, and reproducible workflows, facilitating replication by other regions. While some country-specific SOPs may require clearance before publication, the available materials already provide a strong foundation for adaptation and scale-up. Overall, the ToT program achieved its goal of laying a sustainable foundation for self-reliant genomic surveillance in the region. Participants now act as internal trainers and technical focal points, and in-country training has begun under their leadership. These outcomes showed that long-term training programs are needed to empower local laboratories for independent sequencing and analysis.

## Discussion

This community-led training yielded several practical benefits for public health. First, participant capabilities increased markedly: all trainees self-reported higher confidence in analyzing pathogen sequence data and using bioinformatics tools (Supplementary Figure S2). Many participants have since applied their skills to real-world outbreak investigations and surveillance activities, including the recent Mpox and Ebola outbreaks in East Africa. For instance, the National Public Health Laboratory in Burundi utilized the workflows developed during the training to support the genomic characterization and response to the Mpox outbreak (19, 20). The peer-to-peer structure of the ToT model ensured that these skills were not confined to the initial 12 trainees; instead, they have already been disseminated through in-country training sessions, extending the program's impact across multiple institutions and teams.

Based on our experience, we offer these recommendations for others replicating this model:

- Establish pathogen genomics infrastructure: governments and public health institutions should invest in enhancing computational facilities, reliable internet connectivity, and data storage solutions to support the implementation of bioinformatics into routine public health practices. This effort was only possible through substantial investment in NGS infrastructure and commitment of the Partner States.
- Develop targeted, adaptable capacity-building programs: training programs should address specific regional needs, focusing on smaller, targeted cohorts and adaptable content that accommodates diverse backgrounds. Reproducible workflows and standardized procedures reduce the technical burden on participants, while promoting wider implementation of bioinformatics tools into public health strategy.
- Sustainable funding and support: sustainable capacity-building programs require institutional buy-in and long-term funding from government and international sources (21). However, it is equally important to integrate them into national, regional, and local public health systems. This integration ensures that the initiatives are embedded within the existing health frameworks, allowing for continuous support and alignment with national priorities. This will enable the program to be iterated for new cohorts, including refresher training, and maintain existing capacities. The long-term viability of these efforts will, thus remain a significant challenge unless countries actively participate and support them.
- Policy and strategic alignment with global efforts: the insights from this regional effort demonstrate how local initiatives can be effectively aligned and integrated into broader international genomic surveillance frameworks. Our efforts not only bolster the region's immediate response capabilities but also contribute to a more equitable global network of pathogen genomics expertise, essential for addressing both current and future outbreaks and epidemics (22). In addition, this capacity-building initiative provides a technical foundation for establishing sentinel surveillance in regions heavily affected by epidemic-prone infectious diseases (16, 23). Consequently, national and global health policies should prioritize and expand support for similar initiatives worldwide, as implementing and maintaining genomic surveillance equitable across the globe, is essential for effectively preparing for and responding to future pandemics from the global perspective (24–27).
- Infrastructure and Personnel Requirements: the minimal requirements for establishing foundational genomics surveillance capacity at national level include: one benchtop sequencer (e.g., Illumina iSeq or MiSeq), two bioinformatics-ready laptops or a small server with ≥64 GB RAM, reliable internet access, and cold-chain storage. Personnel requirements include at least one laboratory scientist with molecular biology experience and one bioinformatician with Linux command-line proficiency. Estimated baseline cost for initial setup, excluding consumables, ranges from USD 80,000–100,000 depending on local procurement and infrastructure status. These estimates should guide countries initiating similar efforts.

These programmatic outcomes and lessons have real public health implications. By increasing in-region sequencing and analysis capacity, the EAC's preparedness for future outbreaks is strengthened. Trained laboratories scientists can now locally genotype pathogens and track AMR trends, leading to timelier, data-driven public health actions. Moreover, this case study suggests a generalizable model: other low- and middle-income regions could adapt a multi-phase, locally led ToT approach to build genomics expertise (28, 29). The broader implication is that investing in workforce and systems (rather than only equipment or external services) yields sustainable improvements in disease surveillance and response.

Taken together, these findings support the utility of the ToT program as a scalable model for capacity building. While long-term public health impact will require continued investment and monitoring, short-term institutional uptake has already been demonstrated through concrete applications such as national outbreak sequencing in Burundi and Marburg virus analysis in Tanzania, as well as the successful rollout of in-country trainings in four Partner States. These examples provide early evidence of how trained personnel are translating skills into action, thereby reinforcing the program's potential for replication in other regions.

Furthermore, each of the recommendations proposed in this study is directly informed by the outcomes observed: the call for sustained infrastructure investment is based on the successful deployment of sequencing and computing equipment; the emphasis on small-cohort, targeted training stems from measured proficiency gains; the recommendation for sustainability mechanisms reflects the effectiveness of cascade training and SOP adoption; and the proposal for a regional center of excellence builds on the emerging cross-country network of trained professionals. Together, these elements validate the proposed approach as both grounded in experience and adaptable for future use in comparable settings.

## Conclusions and future directions

Technical and resource constraints remain key challenges for adopting genomic tools in low-resource settings. However, this experience demonstrates that targeted capacity-building through structured, context-specific training programs and sustained institutional support can effectively overcome these barriers. The EAC's ToT program equipped laboratory professionals with actionable bioinformatics and genomics skills, establishing a foundation for sustainable, in-country pathogen surveillance. The cascading training model, combined with investments in infrastructure and tailored curricula, shows that even regions with limited prior capacity can develop robust expertise to support public health needs. This initiative highlights how empowering public health laboratories with genomic tools can transform disease surveillance and outbreak response capabilities. By strengthening regional collaboration and building a network of trained professionals, the program contributes not only to local and regional disease control but also to global health security efforts, aligning with WHO's objectives for a genomic-driven public health response. The lessons learned, particularly in terms of infrastructure readiness, adaptable training approaches, and the importance of embedding capacity within the national health system, provide a practical roadmap for other regions seeking to build similar capabilities.

Future directions include expanding the program to additional countries within the EAC and broadening its scope to cover a broader range of priority pathogens under a One Health framework. Refresher training and in-country sessions will help consolidate skills, ensure practical application, and reinforce sustainability. Continued investment in workforce development, infrastructure, and regional collaboration is critical to maintaining and expanding these capacities beyond the current initiative. A key priority is the establishment of a regional center of excellence for genomic surveillance. This center would provide ongoing technical training, mentorship, and access to shared resources and infrastructure, supporting public health agencies in sustaining their genomics capabilities and facilitating coordinated cross-border outbreak responses. Importantly, the model aligns with Africa CDC's regional strategy for building resilient genomic surveillance networks, emphasizing country ownership, harmonized training, and cross-country collaboration. Strengthening linkages between existing networks will also promote synergistic approaches, optimize resource use, and enhance the region's capacity to detect, respond to, and contain public health threats. Ultimately, this model demonstrates that collaborative capacity-building programs can enable self-reliant and resilient genomic surveillance systems in low-resource settings, thereby contributing to a stronger, more resilient, and equitable global health security architecture.

## Data Availability

The original contributions presented in the study are included in the article/Supplementary material, further inquiries can be directed to the corresponding author.
