# Supplementary material for: A capacity strengthening model toward self-reliant and sustainable one-health workforce in six East African Community Partner States
Source: Front Public Health. 2025 Jul 24;13:1636817. doi: 10.3389/fpubh.2025.1636817 (PMC12328454; doi:10.3389/fpubh.2025.1636817)
Supplement: Supplementary file 1 [file Data_Sheet_1.docx]

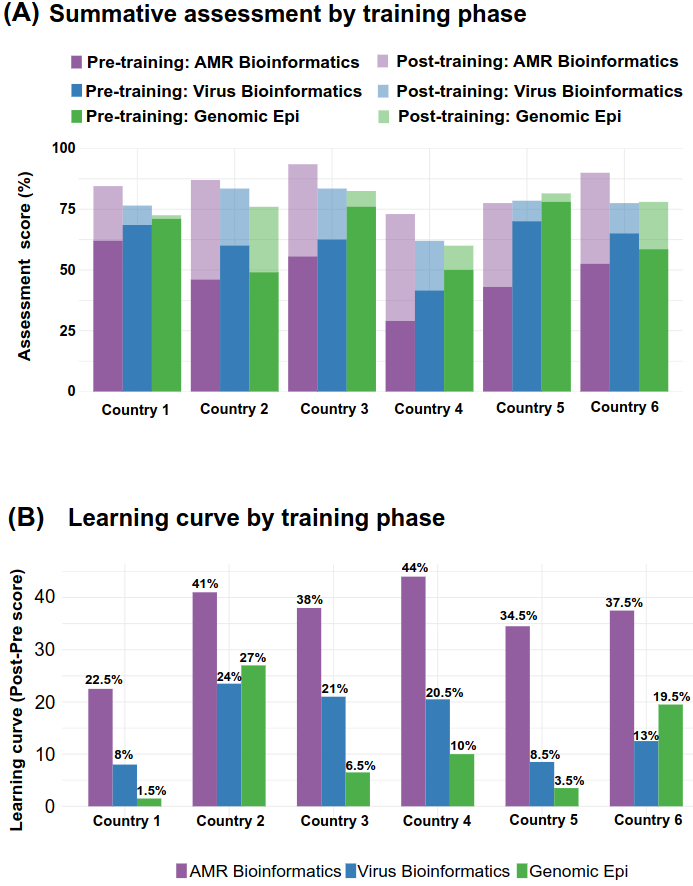


**Supplement Figure S1: Summative assessment of participants' bioinformatics skills by training phase.** (A) Pre- and post-training assessment score for each participating country across the three training phases. (B) Average learning curve (post-training score minus pre-training score) across all participants for each training phase, demonstrating skill acquisition and retention throughout the program.


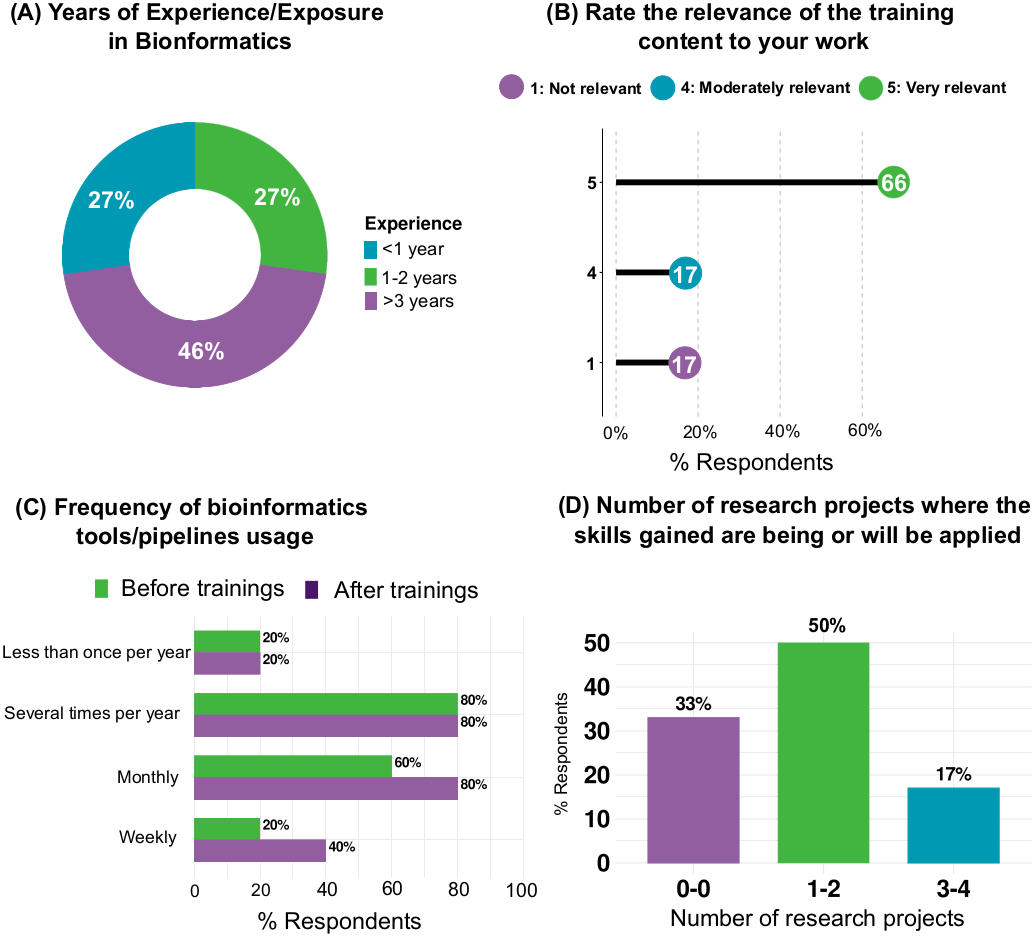


**Supplement Figure S2: Assessment of the training relevance, frequency of tool usage, and application in research projects among participants before and after the ToT program.** (A) Years of experience/exposure to bioinformatics among participants. (B) Participants' rating of the relevance of the training content to their work. (C) Frequency of bioinformatics tools/pipelines usage before and after the training program. (D) Number of research projects where the skills gained are being or will be applied.
